# Efficacy of relacin combined with sodium hypochlorite against *Enterococcus faecalis* biofilms

**DOI:** 10.1590/1678-7757-2016-0608

**Published:** 2018-05-29

**Authors:** CAI Yanling, LIU Hongyan, WEI Xi, CRIELAARD Wim, DENG Dongmei

**Affiliations:** 1 Sun Yat-sen University, Hospital of Stomatology, Guanghua School of Stomatology, Department of Operative Dentistry and Endodontics, Guangzhou, Guangdong, China.; 2 Guangdong Province Key Laboratory of Stomatology, Guangzhou, Guangdong, China.; 3 University of Amsterdam and VU University, Academic Centre for Dentistry Amsterdam (ACTA), Department of Preventive Dentistry, Amsterdam, The Netherlands.; Vrije University, VU University, Academic Centre for Dentistry Amsterdam, Department of Preventive Dentistry,, Amsterdam, Netherlands

**Keywords:** Sodium hypochlorite, Relacin, Enterococcus faecalis, Root canal irrigation, Cytotoxicity

## Abstract

**Objective:**

Relacin is a synthetic molecule that targets RelA, an essential protein in a conserved bacterial stress response system. It was shown to inhibit bacterial growth. The aims of this study were to evaluate the antimicrobial effect of relacin combined with sodium hypochlorite (NaOCl) on *Enterococcus faecalis* biofilms and to evaluate the cytotoxicity of relacin.

**Material and Methods:**

48-h *E. faecalis* OG1RF biofilms were treated by various concentrations of relacin in order to determine its inhibitory concentration. Then, the 48-h biofilms were treated either with 1-min NaOCl (0.01%, 0.05%) alone, or in combination of relacin. As a means of comparison, the biofilms of Δ*relA* were also treated by 1-min NaOCl (0.01%, 0.05%, 0.25%). The treatment efficacy was determined by agar plate count assays. The cytotoxicity of relacin was examined on human gingival epithelial cells Ca9-22 and murine fibroblasts NIH-3T3 by a methyl thiazolyltetrazolium (MTT) assay and a lactate dehydrogenase assay. Statistical analysis was performed by one-way or two-way analysis of variance (ANOVA) with Bonferroni’s *post-hoc* test and an independent Student’s *t*-test. A significance level of p<0.05 was used.

**Results:**

Relacin inhibited the growth of OG1RF biofilms partially at 8 mM and fully at 14 mM. The relacin (14 mM) and NaOCl combined treatment resulted in significantly higher treatment efficacy than NaOCl treatment alone. At 0.05% NaOCl, the combined treatment resulted in 5.65 (±0.19) log reduction in biofilm viability. The Δ*relA* biofilms were more susceptible to NaOCl treatment than the wild type biofilms at 0.25% NaOCl. Relacin at 14 mM was not toxic to host epithelial cells and fibroblasts.

**Conclusions:**

The combination of relacin with a low concentration of NaOCl was effective and not cytotoxic.

## Introduction

Root canal irrigation is a key step to successful root canal therapy. The biological role of irrigation is not only to reduce bacterial infection but also to remove debris in the infected root canal. Sodium hypochlorite (NaOCl) solution has become the most popular irrigant due to its broad antimicrobial spectrum, as well as its unique capacity to dissolve necrotic tissue remnants^29^. So far, there has been no consensus on the clinical concentration of NaOCl. The concentrations vary between 0.5% and 5.25%, depending on the routine of the individual clinic^9^. The irrigation efficacy of NaOCl increases with increasing concentration, but the risk of periapical tissue damage also increases due to the toxicity of NaOCl^5^. The extruding NaOCl into periradicular tissues can cause severe tissue reactions, such as pain, swelling, extensive bruise, and local necrosis^7^. Therefore, a combination of NaOCl with other antimicrobials or surfactants has been suggested to enhance the efficacy of irrigation while maintaining the concentration of NaOCl at a biocompatible level^14^ ^,^ ^23^ ^,^ ^29^.

Several agents have been applied together or in sequence with NaOCl. The combination of 2.5% NaOCl and 0.2% chlorhexidine (CHX), a broad-spectrum antimicrobial, has been shown to be more effective than NaOCl alone^14^. However, the interaction between NaOCl and CHX creates an orange-brown precipitate^4^, which is difficult to remove from the root canal system and may cause discoloration of the dental structures. Alternatively, MTAD, a mixture of doxycycline, citric acid, and a detergent, was shown to inhibit the growth of *Enterococcus faecalis* effectively when applied as the final irrigant after 1.3% NaOCl^18^. However, the same combination was found unable to remove *E. faecalis* in another study^11^. Moreover, there was a concern that bacterial strains in an infected root canal might already be resistant to the doxycycline in MTAD^16^. There is a need for a novel effective agent that can be used together with NaOCl.

It is known that the bacterial species that caused root canal infection can resist nutrient starvation, antibiotics, and other environmental stresses through stringent response (SR)^10^. SR is mediated by intracellular signals like guanosine pentaphosphate or tetraphosphate [(p)ppGpp], and mainly regulated by RelA, a bifunctional enzyme that is able to synthesize and degrade (p)ppGpp^3^. This (p)ppGpp system is one of the essential systems in prokaryotic cells and is highly conserved among various bacterial species^3^. Previous studies^1^ ^,^ ^27^ demonstrated that the (p)ppGpp system was responsible for the resistance of *E. faecalis* to antibiotic treatment, starvation, and oxidative stress. Relacin is a novel compound designed to inhibit the synthetase function of the RelA enzyme, hence reducing the production of (p)ppGpp^25^. A recent study^25^ showed that relacin could function as an antimicrobial agent by impairing the entry of bacterial cells into the stationary phase.

The aim of this study was to evaluate the antimicrobial effect of relacin combined with NaOCl on *E. faecalis* biofilms and to evaluate the cytotoxicity of relacin. Since *E. faecalis* is the predominant microbial species in persistent endodontic infections^30^ and its resistance to stress has been linked to the (p)ppGpp system^1^, this bacterial species was used as a model microorganism in the evaluation. The null hypotheses were that pretreatment of relacin could allow for reduced concentration of NaOCl without comprising its antimicrobial efficacy, and that relacin had no significant cytotoxicity.

## Material and methods

### Bacterial strains and culture conditions

An *E. faecalis* OG1RF strain and its derivative mutant strain Δ*relA* were kindly provided by Professor José A. Lemos^1^. All strains were routinely grown anaerobically (80% N_2_, 10% H_2_, and 10% CO_2_) at 37°C on Brain Heart Infusion (BHI; Difco, Detroit, MI, USA) agar plates.

### Cell lines

Human gingival epithelial cell line Ca9-22 (Japanese Cancer Research Resources Bank, Tokyo, Japan) was cultured in Dulbecco’s Modified Eagle Medium/Nutrient Mixture-F12 with GlutaMax™ (DMEM/F12; Life Technologies, Waltham, MA, USA). Murine fibroblast cell line NIH-3T3 (DSMZ, Braunschweig, Germany) was cultured in Dulbecco’s Modified Eagle Medium (DMEM; Sigma-Aldrich, St. Louis, MO, USA). Both cell culture media were supplemented with 10% fetal bovine serum, 100 U/mL penicillin, 100 μg/mL streptomycin and 0.25 μg/mL amphotericin B. All cells were cultured at 37°C in a humidified atmosphere of 5% CO_2_.

### Chemicals

The relacin was synthesized by Chengdu Chempartner Co. Ltd (Chengdu, Sichuan, China). The structure and composition of relacin was verified by liquid chromatography mass spectrometry and ^1^H-nuclear magnetic resonance^25^. The relacin solution (14 mM) was freshly prepared in bacterial culture medium (BHI broth) or cell culture medium (DMEM/F12 or DMEM) and filter sterilized. NaOCl was purchased from Damao Chemical Reagent Factory (Tianjin, China). The final treatment solutions were prepared freshly in phosphate-buffered saline (PBS).

### Biofilm formation

*E. faecalis* biofilms were grown in a sterile flat-bottom 96-well microtiter plate (Costar, Corning Inc, NY, USA). In detail, a single colony of each *E. faecalis* strain was inoculated into BHI broth for 12 h to reach stationary phase. The preculture was diluted to approximately 5×10^5^ colony forming units (CFU)/mL in fresh BHI broth. The diluted culture (200 μL/well) was transferred into the 96-well microtiter plate and incubated anaerobically for 48 h at 37°C.

### Relacin and NaOCl treatments

All 48-h biofilms were subjected to various treatments after being rinsed once with PBS. At first, the 48-h wild-type biofilms were incubated in 0, 4, 8, and 14 mM relacin solutions for 24 h at 37°C. The concentration that completely inhibited the growth of the biofilms was selected for the following treatments. To compare the treatment efficacy of NaOCl alone or NaOCl combined with relacin, the 48-h wild-type biofilms were treated by NaOCl either directly or after 24-h incubation with relacin (200 μL/well) for 1 min. The concentrations of NaOCl were 0%, 0.01%, and 0.05% (wt/vol). After each treatment, the antimicrobial action of NaOCl was terminated by the removal of treatment solutions and by adding 200 μL of 0.6% sodium thiosulfate to each well^8^.

In addition, the response of Δ*relA* biofilms to NaOCl treatment was compared to that of the wild-type biofilms. To this end, the 48-h Δ*relA* biofilms were treated with 0%, 0.01%, 0.05%, and 0.25% (wt/vol) NaOCl for 1 min as described previously.

Each treatment was tested in triplicate. All experiments were repeated three times.

### Biofilm viability assay

The viability of the biofilm before and after 24 h relacin treatments and of those after NaOCl treatments were examined with the biofilm viability assay.

The biofilms were harvested by scraping and vigorous pipetting. The suspensions were serially diluted and plated onto BHI agar plates. The CFUs were counted after 48 h. CFU counts were transformed to logarithmic values before calculations.

To present the effects of relacin on the growth of *E. faecalis* biofilms, the increases in log CFU per biofilm sample were calculated as the formula: increases of biofilm growth = log_10_ CFU/biofilm (after treatment) – log_10_ CFU/biofilm (before treatment). The killing efficacy of each treatment is presented as the reduction in log CFU per biofilm sample, calculated as the formula: reduction in biofilm viability = log_10_ CFU/biofilm (control sample treated by PBS, 0% NaOCl) – log_10_ CFU/biofilm (treated sample).

### Cytotoxicity assays

The cytotoxicity of relacin was evaluated using Ca9-22 and NIH-3T3 by a 3-(4,5-dimethylthiazol-2-yl)-2,5-diphenyltetrazolium bromide (MTT) and a lactate dehydrogenase (LDH) assays. The cells were seeded into a sterile 96-well tissue-culture plate (Costar, Corning Inc, NY, USA) at a density of 1×10^5^cells/well (100 μL/well) and incubated for 24 h. Thereafter, the culture medium was removed and replaced with 100 μL/well of relacin solution. DMEM/F12 or DMEM (0 mM relacin) was added as a negative control. After a 24-h incubation, supernatants were collected for the evaluation of released lactate dehydrogenase (LDH) activity^28^ and the viability of the cells were examined by the MTT assay. Each group had triplicate wells and the experiment was repeated twice.

For the MTT assay, 10 μL of MTT stock (5 mg/mL) was added to each well and the cells were incubated at 37°C for 2 h. Subsequently, the MTT solution was removed and replaced with 100 μL of dimethyl sulfoxide (DMSO) to dissolve the formazan product. Absorbance of the DMSO was measured by a spectrophotometer (Perkin Elmer, Norwalk, CT, USA) at 570 nm (A_570_). Cell viability was calculated as the formula:

$$\mathrm{cell}\;\mathrm{death}\;(\%)\frac{A_{490}\left(\mathrm{treated}\;\mathrm{group}\right)-A_{490}\left(\mathrm{negative}\;\mathrm{group}\right)}{A_{490}\left(\mathrm{maximum}\;\mathrm{LDH}\;\mathrm{release}\;\mathrm{control}\right)-A_{490}\left(\mathrm{negative}\;\mathrm{control}\right)}\times 100.$$

To quantify LDH activity, 50 μL of supernatants was mixed with equal volume of the assay buffer, containing final concentrations of 0.46 mM 2-piodophenyl-3-p-nitrophenyl-5-phenyl tetrazolium chloride (INT), 0.9 mM nicotinamide adenine dinucleotide (NAD) and 29.7 mM lactic acid. The mixture was incubated for 30 min at room temperature, the absorbance of which was measured at 490 nm (A_490_). The maximum LDH release was obtained by treating the cells with 0.9% (w/v) Triton X-100. The percentage of cell death was calculated using the following formula:

$$\mathrm{cell}\;\mathrm{death}\;(\%)\frac{A_{490}\left(\mathrm{treated}\;\mathrm{group}\right)-A_{490}\left(\mathrm{negative}\;\mathrm{group}\right)}{A_{490}\left(\mathrm{maximum}\;\mathrm{LDH}\;\mathrm{release}\;\mathrm{control}\right)-A_{490}\left(\mathrm{negative}\;\mathrm{control}\right)}\times 100.$$

### Statistical analysis

Statistical analysis was performed with the SPSS 20.0 software. One-way analysis of variance (ANOVA) was used to examine the effect of relacin concentrations on the growth of the biofilms. Two-way ANOVA and Bonferroni’s *post-hoc* test were used to analyze the influence of various combinations of relacin and NaOCl on the treatment efficacy. These analyses were also used to examine the response of wild type and Δ*relA* biofilms to various NaOCl treatments. An independent Student’s *t*-test was applied to analyze the effect of relacin treatment on cell viability and cell death. A significance level of p<0.05 was adopted.

## Results

### Treatment efficacy of relacin and NaOCl

**Figure 1:**
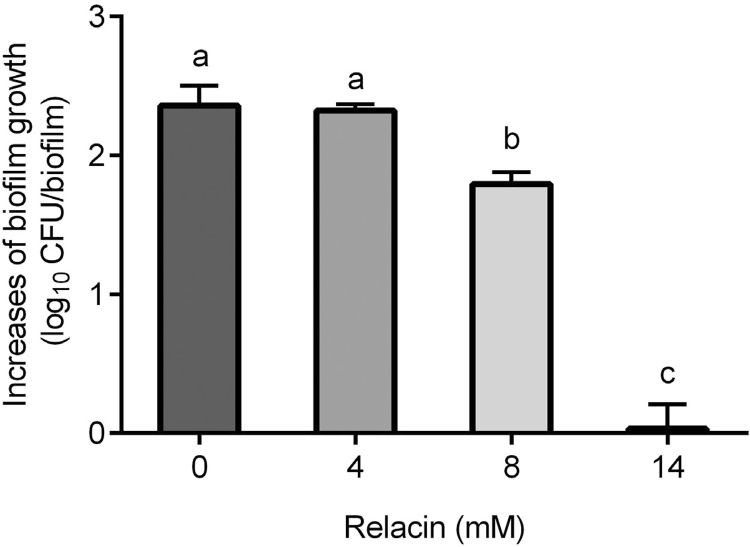
Growth inhibition of *E. faecalis* OG1RF biofilms by relacin treatments for 24 h. The biofilm growth was presented as the log CFU increase in each biofilm sample. Different lowercase letters indicate significant difference (p<0.05)

After 24-h incubation in BHI broth, the number of viable cells in *E. faecalis* wild-type biofilms significantly increased. The addition of relacin inhibited the growth of the biofilms in a dose-dependent manner. The increase in log CFUs of the biofilms in 24 h are showed in Figure 1. Relacin inhibited the biofilm growth partially at 8 mM and fully at 14 mM. Therefore, 14 mM relacin was selected for the following treatments.

**Figure 2:**
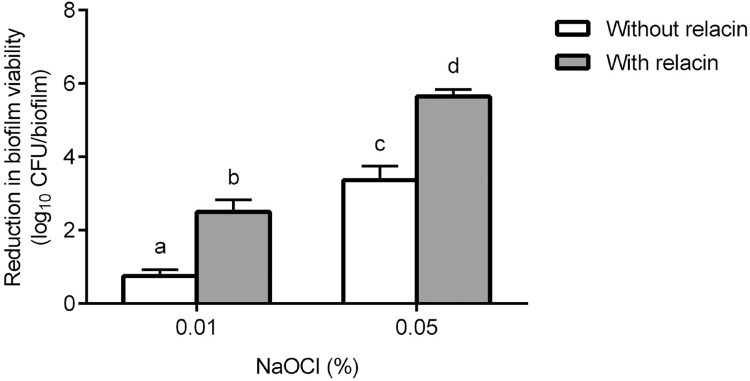
Treatment efficacy of NaOCl alone or in combination with relacin. The 48-h *E. faecalis* OG1RF biofilms were treated by NaOCl alone (white bar) or pretreated by 14 mM relacin for 24 h (gray bar). The efficacy of each treatment is presented as the reduction in biofilm viability. Different lowercase letters indicate significant difference (p<0.05)

The treatment efficacy of NaOCl alone and in combination with 24-h relacin (14 mM) treatment is presented in Figure 2. The treatment efficacy significantly increased with increasing NaOCl concentrations. The additional relacin treatment clearly enhanced the efficacy of NaOCl. At the concentration of 0.05% NaOCl, the combined treatment led to approximately 6-log reduction in biofilm viability.

### Response of *E. faecalis* Δ*relA* biofilms to NaOCl

**Figure 3:**
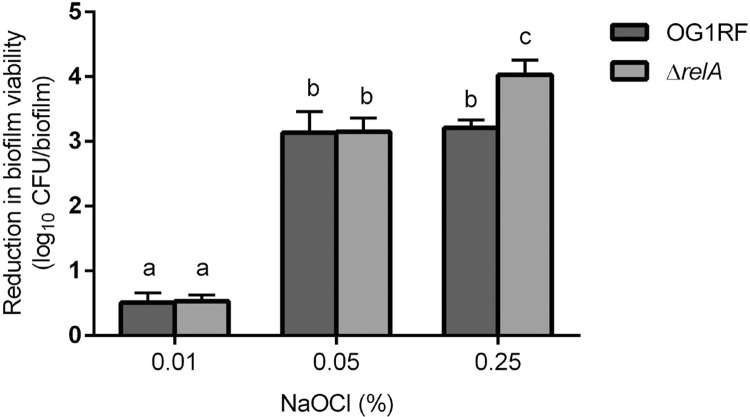
Treatment efficacy of NaOCl on *E. faecalis* biofilms. The 48-h *E. faecalis* OG1RF (dark gray bar) and Δ*relA* (gray bar) biofilms were treated by NaOCl. The efficacy of each treatment is presented as the reduction in biofilm viability. Different lowercase letters indicate significant difference (p<0.05)

As shown in Figure 3, NaOCl treatments resulted in a dose-dependent reduction in biofilm viability, irrespective of the strains tested. Significantly higher reduction was observed in Δ*relA*biofilms than in the wild type biofilms, when NaOCl concentration was 0.25%.

### Cytotoxicity of relacin

**Figure 4:**
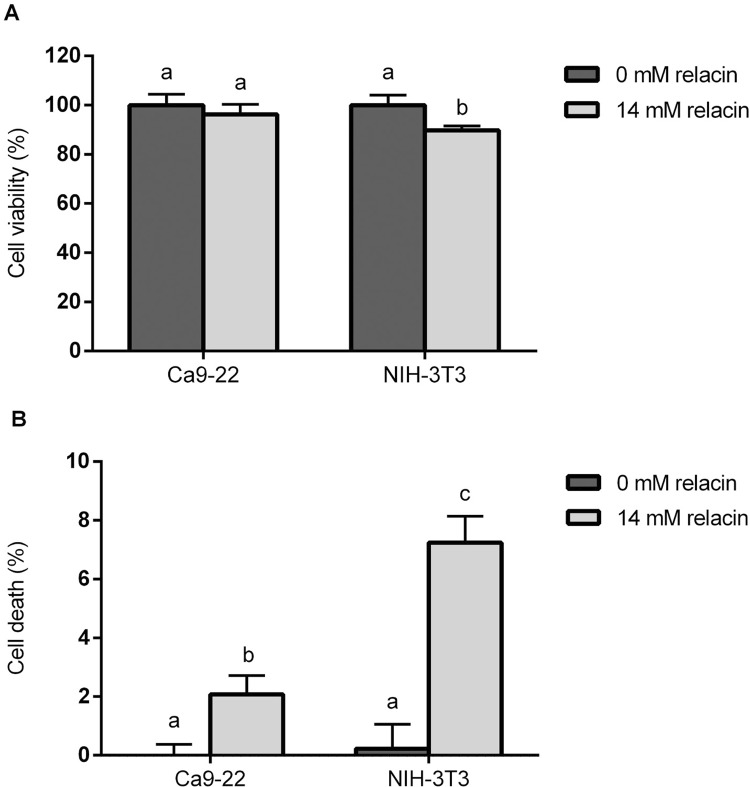
Effect of relacin treatment on Ca9-22 and NIH-3T3 cells. The confluent Ca9-22 or NIH-3T3 cells were treated with 14 mM relacin or culture medium (0 mM relacin) for 24 h. The cell viability was assessed by MTT assay (A) and the cell death was examined by measuring the LDH activity in extracellular culture medium (B). Different lowercase letters indicate significant difference in each cell line (p<0.05)

The MTT readings showed that the viability of epithelial cells Ca9-22 was not affected by the 24-h treatment with 14 mM relacin. In addition, the viability of fibroblasts NIH-3T3 was hardly reduced (approximately 10% reduction) (Figure 4A). Similar findings could be found using the LDH release assay (Figure 4B). After the relacin treatment, a low percentage of cell death was observed for Ca9-22 (2.08 % ± 0.65) and NIH-3T3 (7.24% ± 0.90), respectively.

## Discussion

This study showed that the 1-min application of 0.05% NaOCl after 14 mM relacin pretreatment resulted in approximately 6-log reduction in the viability of *E. faecalis* biofilms. Relacin at 14 mM did not exhibit obvious cytotoxicity to host cells. Therefore, the combined treatment can reduce the potential cytotoxicity of NaOCl by lowering its concentration and application time^24^, without compromising the antimicrobial efficacy of NaOCl.

It was hypothesized that relacin is not harmful for human tissue because relacin is designed to bind specifically to the synthetase site of a microbial RelA protein, which is not present in the eukaryotic cells^21^. The data of this study supports this hypothesis. In this study, the cytotoxicity of relacin was evaluated by using two types of host cells, gingival epithelial cells and fibroblasts, which represent the major cell types in oral mucosa and periradicular tissue. The cytotoxicity was examined with two types of assays, MTT and LDH. Both assays were used to assess the biosafety of novel dental materials or treatments. MTT reduction is a marker reflecting viable cell metabolism, while the detection of LDH activity in the extracellular medium indicates irreversible cell death due to cell membrane damage^6^. This study showed that the results from these two assays were in line with each other and that the overall cytotoxicity of relacin was low. Collectively, relacin itself is harmless to the host cells and it can improve the safety of NaOCl by reducing its treatment concentration. Relacin is a promising candidate to be used together with NaOCl.

Our data showed that the pre-treatment of relacin reduced the effective concentration of NaOCl to 0.05%. This result is comparable to what was reported for the combination of CHX/calcium hydroxide and NaOCl. After the pre-treatment of chlorhexidine or calcium hydroxide, a minimum of 1% NaOCl was needed for 3 to 6-log reduction in bacterial cell counts^20^ ^,^ ^23^. The enhanced killing efficacy of NaOCl after relacin treatment may be explained by inhibition of the (p)ppGpp system. It is likely that relacin inactivated RelA by binding to the synthetase site of the enzyme, hence inhibiting the production of (p)ppGpp^25^. As a result, *E. faecalis* biofilms became vulnerable to oxidative stress. This hypothesis can be supported by previous findings about RelA being required for *E. faecalis* biofilms to resist NaOCl^1^ ^,^ ^27^, and by this data on Δ*relA* biofilms. In this proof-of-principal study, the efficacy of 14 mM relacin was tested, since it could fully inhibit the growth of *E. faecalis* biofilms. In future studies, it will be examined if similar efficacy could be achieved with lower concentration of relacin. Unlike common antimicrobial agents, relacin inhibits the growth of bacterial cells and makes them vulnerable to stress rather than kill the cells^27^. This mode of action requires a relatively long application time — in this case, 24 h. Clinically, relacin may be used as an intracanal medicament before NaOCl irrigation.

It is known that root canal infection is caused by multiple bacterial species. Microorganisms such as *Enterococcus faecium*, *Pseudoramibacter alactolyticus,* and *Fusobacterium nucleatum* have also been reported to be associated with the infections^17^ ^,^ ^22^. It could be valuable for the clinic if the combined treatment was also effective against other bacterial species aside from *E. faecalis*. Since nearly all bacterial species examined so far can induce SR through the signaling molecule (p)ppGpp, mainly regulated by RelA proteins^10^, it is very likely that relacin exhibits similar effect on other bacterial species. Such potential will be explored in further study.

The biofilm model used in this study may have the limitations of biofilm age and absence of dentin. Currently, different substrates have been used to develop bacterial biofilms such as dentin, polystyrene culture plates, cellulose nitrate membrane filters and hydroxyapatite discs, among which polystyrene culture plates were commonly used in *in vitro* experiments for offering rapid retrieval and quantification of biofilms^12^. Several studies^2^ ^,^ ^13^ showed that *E. faecalis* could develop matured biofilms after incubation of 20 h on glass rods or 24 h on polystyrene pegs. Moreover, Kristich, et al.^13^ (2004) demonstrated that the biofilm development of *E. faecalis* was maintained at a constant level from 24 h to 48 h in the wells of microtiter plates, which can establish a quantifiable biofilm. Other studies^19^ ^,^ ^26^ had used 48-h biofilms of *E. faecalis* to evaluate the antibacterial effects of antibiotics and intracanal medicaments in polystyrene plates. The previous study^15^ also indicated that *E. faecalis* was able to form matured biofilms after incubation for 48 h on polystyrene blocks. Accordingly, in this study, a 48-h *E. faecalis* biofilms can be applied for the antimicrobial assays. However, dentin may be much closer to the clinical situation^12^. Therefore, further study could be performed on the dentin with different ages of biofilms. Despite the limitations of the biofilm model, the data of this study can support that relacin has the potential to allow reduced concentration of NaOCl without comprising its antimicrobial efficacy.

## Conclusions

The data of this study demonstrated that the application of relacin allowed reduced concentrations of NaOCl without comprising its antimicrobial efficacy. The combination of relacin with a low concentration of NaOCl was effective and not cytotoxic.
